# CT OF THE ENDOCRANIUM IN THE FIRST EPISODES OF PSYCHOTIC DISORDERS

**DOI:** 10.1192/j.eurpsy.2023.2210

**Published:** 2023-07-19

**Authors:** A. Tomcuk, J. Dedovic, A. Macic

**Affiliations:** Special Psychiatric Hospital Kotor, Kotor, Montenegro

## Abstract

**Introduction:**

CT of the endocranium is an indispensable step in the differential-diagnostic evaluation of a patient with the first episode of psychotic disorder. In this context, the main goal of this diagnostic procedure is to “exclude” the so-called organic disorders of the brain tissue, which could be manifested by psychotic phenomenology. However, during the past decades, pathological changes in the brain parenchyma have been found during neuroimaging studies of psychiatric patients.

With the group of schizophrenic psychoses, the following are most often associated: Expansion of the ventricular system, cortical reductive changes, agenesis of the corpus callosum, a higher frequency of some congenital anomalies of the brain, such as cavum septum pellucidum and cavum verga.

**Objectives:**

Consequently, the main goal of our study is to demonstrate the possibility of diagnosing these changes and examine the frequency of cases of so-called secondary psychotic syndromes, where psychotic phenomenology arose on the basis of some other illness.

**Methods:**

Data were collected for 145 patients from the Acute Male Department, who were treated in the period 2011-2019. CT scan of the endocranium was performed as part of the diagnostic evaluation during the first psychiatric hospitalization.

**Results:**

Out of the total number of examinations (145), 20.7% of patients had a pathological finding, with the following structure: In 6.9% of the age group up to 45 years, moderately expressed (disproportionate for the age) cortical reductive changes were found. Concomitant occurrence of cavum septi pellucid et cavum vergae was observed in 3.4% and isolated septum pellucidum cyst in 1.3% of patients. Post-stroke changes, post-contusion foci, and benign congenital brain tumors were found with a frequency of 1.3%, pronounced cortical reductive changes and pathological calcifications with a frequency of 3%, while in individual cases (0.68%) the presence of a subarachnoid cyst and atrophy of the cerebellar cortex was recorded

**Conclusions:**

CT of the endocranium represents a significant aid in the evaluation of a patient with the first episode of psychotic disorder. However, its use in order to analyze more complex changes in psychiatric patients is significantly limited compared to more modern neuroimaging modalities.

**Disclosure of Interest:**

None Declared

